# Supplementary material for: Root Ethylene and Abscisic Acid Responses to Flooding Stress in Styrax japonicus: A Transcriptomic Perspective
Source: Plants (Basel). 2025 Jun 18;14(12):1870. doi: 10.3390/plants14121870 (PMC12197084; doi:10.3390/plants14121870)
Supplement: Supplementary file 1 [file plants-14-01870-s001.zip › plants-3552591-supplementary.pdf]

Supplementary Materials:

Supplementary figure S1 qRT-PCR verification of 8 DEGs. The bar chart indicates the relative expression level, and the dot plot indicates the TPM value. Light blue represents samples under waterlogging stress and dark blue represents samples under submergence.

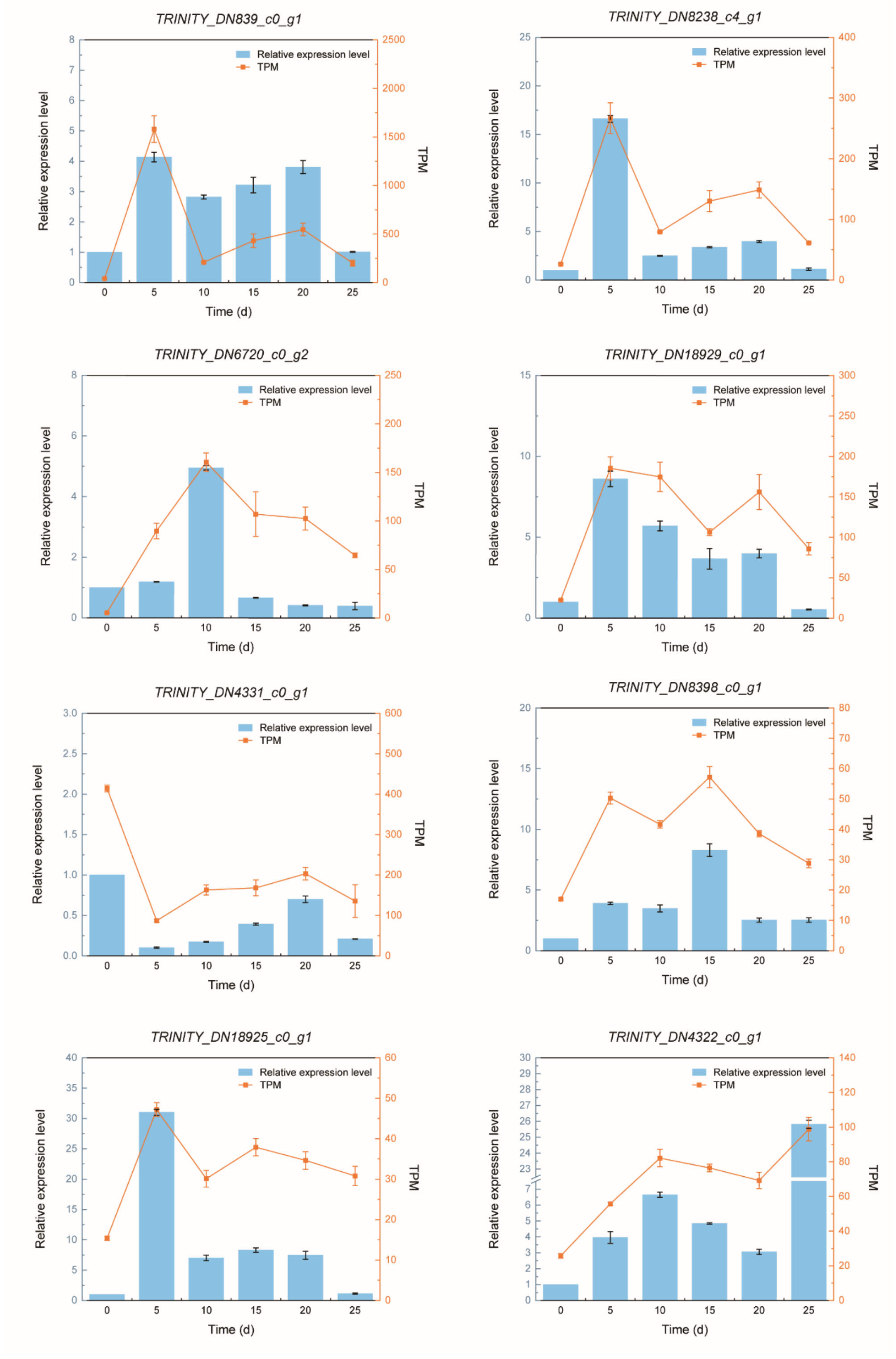

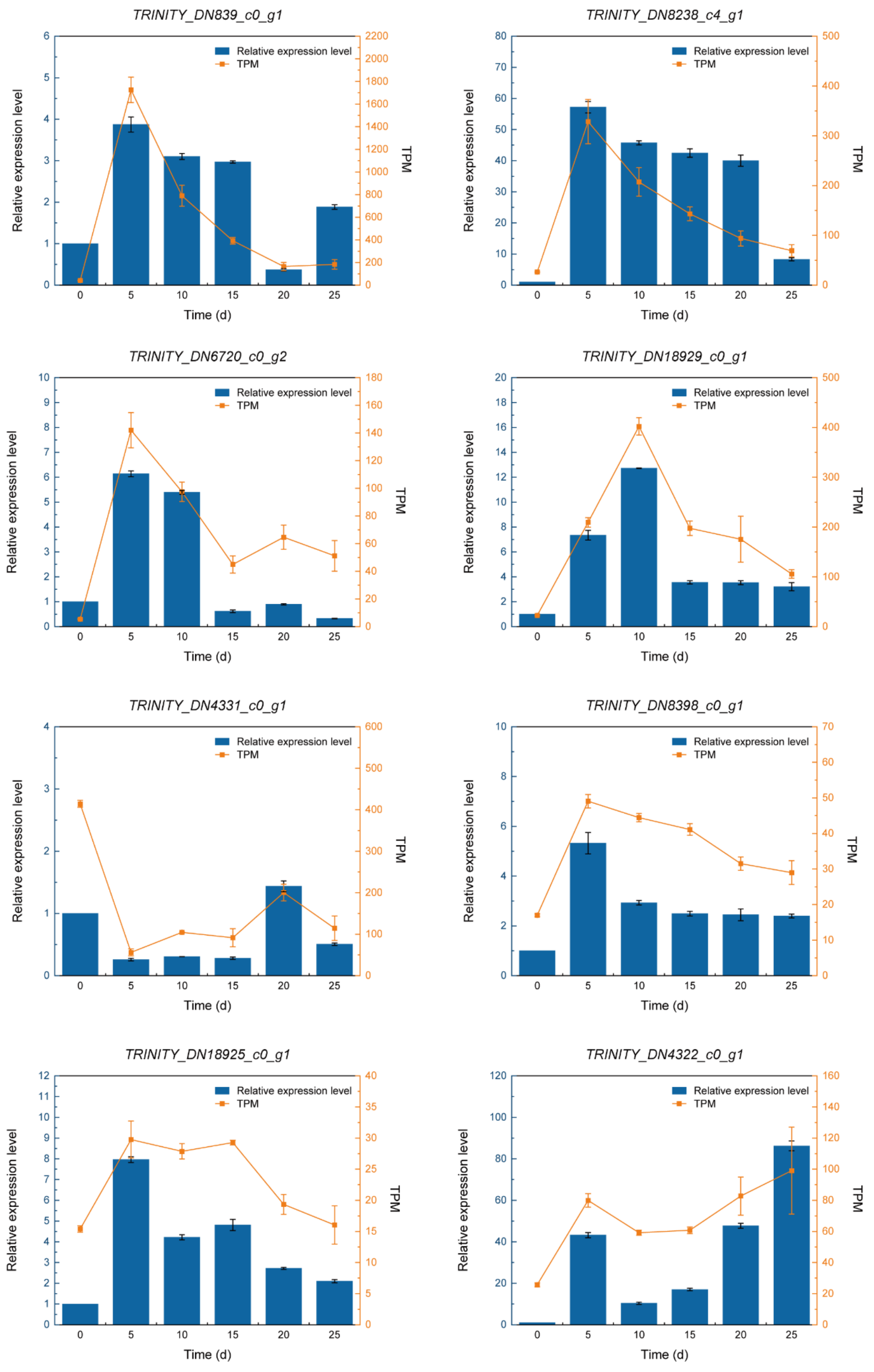

TRINITY_DN839_c0_g1
TRINITY_DN8238_c4_g1
TRINITY_DN6720_c0_g2
TRINITY_DN18929_c0_g1
TRINITY_DN4331_c0_g1
TRINITY_DN8398_c0_g1
TRINITY_DN18925_c0_g1
TRINITY_DN4322_c0_g1
Relative expression level
TPM
Time (d)

Supplementary table S1 Table of sequencing and quality control

Sample	Raw reads	Raw bases	Valid reads	Valid bases	Valid %	Q20 %	Q30 %	GC %
0d1	58362250	8.75G	57591618	8.55G	98.68	98.83	96.23	45.49
0d2	40996392	6.15G	40247984	5.95G	98.17	98.62	95.69	45.74
0d3	54699586	8.20G	53957616	8.01G	98.64	98.77	96.04	45.52
Z_5d1	45346246	6.80G	44386992	6.56G	97.88	98.61	95.79	46.34
Z_5d2	43231570	6.48G	42253782	6.23G	97.74	98.63	95.88	45.77
Z_5d3	43691534	6.55G	42555640	6.27G	97.40	98.63	95.88	46.62
Z_10d1	46713036	7.01G	45764668	6.76G	97.97	98.65	95.87	45.99
Z_10d2	46659182	7.00G	45691218	6.75G	97.93	98.69	96.00	46.06
Z_10d3	44680534	6.70G	43790972	6.47G	98.01	98.66	95.88	46.14
Z_15d1	46596850	6.99G	45658124	6.75G	97.99	98.63	95.89	46.55
Z_15d2	44660620	6.70G	43653032	6.44G	97.74	98.62	95.80	46.83
Z_15d3	44457012	6.67G	43533266	6.43G	97.92	98.60	95.78	46.90
Z_20d1	45495742	6.82G	44567890	6.58G	97.96	98.63	95.85	46.71
Z_20d2	46431464	6.96G	45483842	6.72G	97.96	98.66	95.90	46.88
Z_20d3	41457358	6.22G	40648408	6.00G	98.05	98.58	95.71	46.81
Z_25d1	45610508	6.84G	44656124	6.60G	97.91	98.63	95.81	46.82
Z_25d2	43389388	6.51G	42237036	6.23G	97.34	98.68	95.96	47.09
Z_25d3	48054888	7.21G	46795816	6.90G	97.38	98.68	95.95	46.51
L_5d1	44448958	6.67G	43572394	6.44G	98.03	98.67	95.96	46.24
L_5d2	45847632	6.88G	44872996	6.63G	97.87	98.68	95.96	46.10
L_5d3	43416928	6.51G	42570562	6.29G	98.05	98.69	95.96	46.28
L_10d1	42001704	6.30G	40998812	6.05G	97.61	98.60	95.80	46.52
L_10d2	44094302	6.61G	43224300	6.39G	98.03	98.63	95.80	46.18
L_10d3	44553180	6.68G	43630700	6.45G	97.93	98.65	95.87	46.25
L_15d1	44703208	6.71G	43741792	6.46G	97.85	98.65	95.89	47.01
L_15d2	46863520	7.03G	45851022	6.77G	97.84	98.68	95.98	46.49
L_15d3	44826512	6.72G	43823554	6.47G	97.76	98.57	95.70	46.76
L_20d1	40289748	6.04G	39386506	5.81G	97.76	98.66	95.86	46.76
L_20d2	40921744	6.14G	40117806	5.93G	98.04	98.64	95.79	47.15
L_20d3	46983722	7.05G	46015982	6.80G	97.94	98.63	95.85	46.57
L_25d1	50036998	7.51G	48654406	7.17G	97.24	98.58	95.76	50.84
L_25d2	46217424	6.93G	44830414	6.60G	97.00	98.68	96.02	47.84
L_25d3	49469854	7.42G	47949300	7.06G	96.93	98.63	95.81	48.13

Supplementary table S2 A total of 54 common DEGs across the five sample groups in the plant hormone signal transduction pathway under waterlogging stress

Gene id	Annotation	Name
TRINITY_DN3134_c1_g2	homeodomain-like superfamily protein [*Actinidia rufa*]	BOA
TRINITY_DN32741_c0_g1	Ethylene-responsive transcription factor 1B like [Actinidia chinensis var. chinensis]	ERF.C.3
TRINITY_DN3702_c0_g1	hypothetical protein F0562_029054 [*Nyssa sinensis*]	-
TRINITY_DN37886_c0_g1	protein SMALL AUXIN UP-REGULATED RNA 51-like [*Humulus lupulus*]	SAUR50
TRINITY_DN38841_c0_g1	hypothetical protein TEA_028362 [*Camellia sinensis* var. sinensis]	SAPK10
TRINITY_DN24089_c0_g1	hypothetical protein HYC85_009813 [*Camellia sinensis*]	-
TRINITY_DN24406_c0_g1	AUX/IAA transcriptional regulator family protein [*Actinidia rufa*]	AUX22D
TRINITY_DN12242_c1_g1	putative protein phosphatase 2C 6 [*Camellia lanceoleosa*]	ABI1
TRINITY_DN12427_c0_g1	hypothetical protein QN277_009698 [*Acacia crassicarpa*]	BAK1
TRINITY_DN12701_c0_g2	ethylene-response factor C3-like [*Actinidia eriantha*]	ERF.C.3
TRINITY_DN131348_c0_g1	Histidine-containing phosphotransfer protein 1 [*Camellia lanceoleosa*]	AHP1
TRINITY_DN13551_c0_g1	hypothetical protein RGQ29_006622 [*Quercus rubra*]	XTH23
TRINITY_DN13721_c0_g1	uncharacterized protein LOC114319691 [*Camellia sinensis*]	-
TRINITY_DN76305_c0_g1	protein DOG1-like 4 [Camellia sinensis]	DOGL4

Gene id	Annotation	Name
TRINITY_DN8160_c0_g1	jasmonate ZIM-domain 7 [*Camellia sinensis*]	-
TRINITY_DN8459_c0_g1	PREDICTED: protein AUXIN SIGNALING F-BOX 2-like [*Nelumbo nucifera*]	AFB2
TRINITY_DN8680_c1_g1	Serine/threonine-protein kinase BSK5 [*Camellia lanceoleosa*]	BSK5
TRINITY_DN5917_c1_g1	Auxin response factor 4 [*Camellia lanceoleosa*]	ARF4
TRINITY_DN6457_c0_g1	hypothetical protein RHMOL_Rhmol02G0039100 [*Rhododendron molle*]	ERF095
TRINITY_DN6720_c0_g2	Abscisic acid receptor PYL4 [*Camellia lanceoleosa*]	PYL4
TRINITY_DN6720_c0_g3	abscisic acid receptor PYL4-like [*Camellia sinensis*]	PYL4
TRINITY_DN6720_c1_g1	abscisic acid receptor PYL4-like [*Diospyros lotus*]	-
TRINITY_DN185719_c0_g3	GRAS family protein RAD1-like [*Diospyros lotus*]	RAD1
TRINITY_DN18929_c0_g1	hypothetical protein HHK36_025218 [*Tetracentron sinense*]	SAUR51
TRINITY_DN18938_c0_g1	hypothetical protein F0562_029163 [*Nyssa sinensis*]	PIL15
TRINITY_DN20260_c0_g1	histidine kinase 2 isoform X1 [*Actinidia eriantha*]	-
TRINITY_DN2036_c0_g1	probable protein phosphatase 2C 24 isoform X1 [*Actinidia eriantha*]	SAG113
TRINITY_DN2036_c0_g2	probable protein phosphatase 2C 24 [*Nicotiana tomentosiformis*]	At2g29380
TRINITY_DN21163_c0_g1	Ethylene-responsive transcription factor 1B like [*Actinidia chinensis* var. chinensis]	ERF.C.3

Gene id	Annotation	Name
TRINITY_DN21710_c0_g1	ABSCISIC ACID-INSENSITIVE 5-like protein [*Actinidia chinensis* var. chinensis]	ABF4
TRINITY_DN23610_c0_g1	hypothetical protein F0562_033809 [*Nyssa sinensis*]	PHO1;H3
TRINITY_DN26946_c0_g1	hypothetical protein RHGRI_020045 [*Rhododendron griersonianum*]	SCL28
TRINITY_DN27373_c0_g1	ethylene-response factor C3-like [*Cornus florida*]	ERF1B
TRINITY_DN2842_c0_g2	ethylene receptor 2-like [*Camellia sinensis*]	ETR2
TRINITY_DN10100_c0_g1	hypothetical protein RHMOL_Rhmol03G0177000 [*Rhododendron molle*]	ARR17
TRINITY_DN1048_c0_g2	serine/threonine-protein kinase BSK5-like [*Diospyros lotus*]	BSK5
TRINITY_DN105649_c0_g1	hypothetical protein RHGRI_036335 [*Rhododendron griersonianum*]	SCL9
TRINITY_DN10573_c0_g1	transcription factor MYC2-like [*Diospyros lotus*]	MYC2
TRINITY_DN108595_c0_g1	scarecrow-like protein 14 [*Actinidia eriantha*]	SCL14
TRINITY_DN10950_c0_g1	Phosphate transporter PHO1 [*Camellia lanceoleosa*]	PHO1
TRINITY_DN10959_c0_g1	hypothetical protein CISIN_1g023816mg [*Citrus sinensis*]	ARR4
TRINITY_DN9951_c0_g1	Auxin response factor 9 [*Camellia lanceoleosa*]	ARF9
TRINITY_DN4322_c0_g1	auxin-responsive protein SAUR71-like [*Camellia sinensis*]	SAUR72

**Gene id**	**Annotation**	**Name**
TRINITY_DN51052_c0_g1	hypothetical protein PVAP13_7NG419100 [*Panicum virgatum*]	DDB_G0282895
TRINITY_DN5172_c0_g2	Ethylene-responsive transcription factor 1B like [*Actinidia chinensis* var. chinensis]	ERF.C.3
TRINITY_DN5386_c0_g1	hypothetical protein RHSIM_Rhsim03G0226700 [*Rhododendron simsii*]	TIFY10A
TRINITY_DN16520_c0_g1	Protein TIFY like [*Actinidia chinensis* var. chinensis]	TIFY9
TRINITY_DN17431_c2_g1	Serine/threonine-protein kinase SAPK10 [*Ananas comosus*]	SRK2E
TRINITY_DN177750_c0_g1	protein DOG1-like 4 [*Camellia sinensis*]	DOGL4
TRINITY_DN18329_c0_g1	hypothetical protein GOBAR_DD09366 [*Gossypium barbadense*]	BAK1
TRINITY_DN1446_c1_g1	BRASSINOSTEROID INSENSITIVE 1-associated receptor kinase 1-like [*Salvia hispanica*]	SERK2
TRINITY_DN1446_c5_g1	BRASSINOSTEROID INSENSITIVE 1-associated receptor kinase 1 [*Rosa chinensis*]	BAK1
TRINITY_DN14517_c0_g1	ethylene-responsive transcription factor 1B-like [*Camellia sinensis*]	ERF1B
TRINITY_DN15733_c0_g1	Transcription factor HHO3 [*Sesamum alatum*]	HHO3

Supplementary table S3 A total of 112 common DEGs across the five sample groups in the plant hormone signal transduction pathway under submergence

Gene id	Annotation	Name
TRINITY_DN304_c0_g1	auxin-responsive protein IAA14-like [*Diospyros lotus*]	IAA7
TRINITY_DN3134_c1_g2	homeodomain-like superfamily protein [*Actinidia rufa*]	BOA
TRINITY_DN32741_c0_g1	Ethylene-responsive transcription factor 1B like [*Actinidia chinensis* var. chinensis]	ERF.C.3
TRINITY_DN35124_c0_g1	basic helix-loop-helix transcription factor [*Loropetalum chinense* var. rubrum]	MYC2
TRINITY_DN3536_c0_g1	auxin-responsive GH3 family protein [*Actinidia rufa*]	GH3.1
TRINITY_DN3702_c0_g1	hypothetical protein F0562_029054 [*Nyssa sinensis*]	-
TRINITY_DN3738_c0_g1	hypothetical protein RHGRI_023426 [*Rhododendron griersonianum*]	At5g66900
TRINITY_DN3771_c0_g1	Auxin-responsive protein SAUR36 [*Camellia lanceoleosa*]	SAUR36
TRINITY_DN3782_c0_g1	histidine-containing phosphotransfer protein 1-like isoform X1 [*Actinidia eriantha*]	AHP1
TRINITY_DN37886_c0_g1	protein SMALL AUXIN UP-REGULATED RNA 51-like [*Humulus lupulus*]	SAUR50
TRINITY_DN38841_c0_g1	hypothetical protein TEA_028362 [*Camellia sinensis* var. sinensis]	SAPK10
TRINITY_DN23827_c0_g1	hypothetical protein CCACVL1_17955 [*Corchorus capsularis*]	LSH1
TRINITY_DN23832_c0_g1	auxin transporter-like protein 2 [*Camellia sinensis*]	LAX5
TRINITY_DN2393_c1_g1	Protein TIFY 6B [*Camellia lanceoleosa*]	TIFY6B
TRINITY_DN24089_c0_g1	hypothetical protein HYC85_009813 [*Camellia sinensis*]	-

Gene id	Annotation	Name
TRINITY_DN241003_c0_g1	PREDICTED: serine/threonine-protein kinase HT1-like [*Fragaria vesca* subsp. vesca]	yes1
TRINITY_DN24607_c0_g1	hypothetical protein Vadar_006304 [*Vaccinium darrowii*]	SCL14
TRINITY_DN248875_c0_g1	hypothetical protein Vadar_000561 [*Vaccinium darrowii*]	XTH25
TRINITY_DN26040_c0_g1	auxin-responsive protein SAUR71-like [*Alnus glutinosa*]	-
TRINITY_DN12366_c0_g1	two-component response regulator ORR9-like [*Camellia sinensis*]	RR4
TRINITY_DN1236_c0_g1	protein TIFY 10b [*Actinidia eriantha*]	TIFY10A
TRINITY_DN12427_c0_g1	hypothetical protein QN277_009698 [*Acacia crassicarpa*]	BAK1
TRINITY_DN12701_c0_g2	ethylene-response factor C3-like [*Actinidia eriantha*]	ERF.C.3
TRINITY_DN12949_c0_g1	auxin response factor 18-like [*Camellia sinensis*]	ARF9
TRINITY_DN131348_c0_g1	Histidine-containing phosphotransfer protein 1 [*Camellia lanceoleosa*]	AHP1
TRINITY_DN13551_c0_g1	hypothetical protein RGQ29_006622 [*Quercus rubra*]	XTH23
TRINITY_DN13721_c0_g1	uncharacterized protein LOC114319691 [*Camellia sinensis*]	-
TRINITY_DN7344_c0_g2	Transcription factor TGA1 [*Camellia lanceoleosa*]	TGA3
TRINITY_DN76305_c0_g1	protein DOG1-like 4 [*Camellia sinensis*]	DOGL4
TRINITY_DN8074_c0_g1	scarecrow-like transcription factor PAT1 [*Camellia sinensis*]	PAT1
TRINITY_DN8414_c0_g1	BRASSINOSTEROID INSENSITIVE 1-associated receptor kinase [*Sesamum angolense*]	BAK1

Gene id	Annotation	Name
TRINITY_DN8459_c0_g1	PREDICTED: protein AUXIN SIGNALING F-BOX 2-like [*Nelumbo nucifera*]	AFB2
TRINITY_DN8588_c0_g1	auxin response factor 6-like isoform X1 [*Actinidia eriantha*]	ARF6
TRINITY_DN859_c0_g1	unnamed protein product [*Closterium* sp. Naga37s-1]	NIK1
TRINITY_DN87489_c0_g1	Histidine kinase [*Actinidia chinensis* var. chinensis]	AHK5
TRINITY_DN5813_c0_g1	Carboxylesterase 1 [*Camellia lanceoleosa*]	CXE1
TRINITY_DN5917_c1_g1	Auxin response factor 4 [*Camellia lanceoleosa*]	ARF4
TRINITY_DN61913_c0_g1	DELLA protein RGL2 [*Camellia lanceoleosa*]	SCL23
TRINITY_DN6457_c0_g1	hypothetical protein RHMOL_Rhmol02G0039100 [*Rhododendron molle*]	ERF095
TRINITY_DN64728_c0_g2	hypothetical protein Vadar_026602 [*Vaccinium darrowii*]	CXE6
TRINITY_DN6526_c0_g1	protein SHORT-ROOT [*Vitis riparia*]	SHR
TRINITY_DN6720_c0_g2	Abscisic acid receptor PYL4 [*Camellia lanceoleosa*]	PYL4
TRINITY_DN6720_c0_g3	abscisic acid receptor PYL4-like [*Camellia sinensis*]	PYL4
TRINITY_DN6720_c1_g1	abscisic acid receptor PYL4-like [*Diospyros lotus*]	-
TRINITY_DN185719_c0_g3	GRAS family protein RAD1-like [*Diospyros lotus*]	RAD1
TRINITY_DN186416_c0_g1	Auxin response factor 4 [*Camellia lanceoleosa*]	ARF4
TRINITY_DN18691_c0_g1	hypothetical protein Acr_27g0007650 [*Actinidia rufa*]	SCL3
TRINITY_DN18929_c0_g1	hypothetical protein HHK36_025218 [*Tetracentron sinense*]	SAUR51
TRINITY_DN18938_c0_g1	hypothetical protein F0562_029163 [*Nyssa sinensis*]	PIL15
TRINITY_DN18938_c0_g2	transcription factor PIF3-like [*Actinidia eriantha*]	PIL15

Gene id	Annotation	Name
TRINITY_DN1910_c0_g1	hypothetical protein F0562_034879 [*Nyssa sinensis*]	ARR1
TRINITY_DN19620_c0_g1	Auxin response factor like [*Actinidia chinensis* var. chinensis]	ARF2A
TRINITY_DN200139_c0_g1	hypothetical protein F0562_015897 [*Nyssa sinensis*]	CXE15
TRINITY_DN20260_c0_g1	histidine kinase 2 isoform X1 [*Actinidia eriantha*]	-
TRINITY_DN2036_c0_g2	probable protein phosphatase 2C 24 [*Nicotiana tomentosiformis*]	At2g29380
TRINITY_DN20418_c0_g1	GRAS family protein RAM1-like [*Actinidia eriantha*]	RAM1
TRINITY_DN2094_c0_g1	LOW QUALITY PROTEIN: basic helix-loop-helix protein A-like [*Camellia sinensis*]	BHLH
TRINITY_DN21163_c0_g1	Ethylene-responsive transcription factor 1B like [*Actinidia chinensis* var. chinensis]	ERF.C.3
TRINITY_DN21710_c0_g1	ABSCISIC ACID-INSENSITIVE 5-like protein [*Actinidia chinensis* var. chinensis]	ABF4
TRINITY_DN22212_c0_g1	hypothetical protein HHK36_016386 [*Tetracentron sinense*]	SCL14
TRINITY_DN23610_c0_g1	hypothetical protein F0562_033809 [*Nyssa sinensis*]	PHO1;H3
TRINITY_DN2365_c0_g2	scarecrow-like protein 14 [*Actinidia eriantha*]	SCL14
TRINITY_DN26910_c1_g1	hypothetical protein HYC85_009720 [*Camellia sinensis*]	CYCD3-1
TRINITY_DN26946_c0_g1	hypothetical protein RHGRI_020045 [*Rhododendron griersonianum*]	SCL28
TRINITY_DN27041_c0_g2	auxin-responsive protein IAA13-like [*Camellia sinensis*]	IAA13
TRINITY_DN27373_c0_g1	ethylene-response factor C3-like [*Cornus florida*]	ERF1B

Gene id	Annotation	Name
TRINITY_DN2842_c0_g2	ethylene receptor 2-like [*Camellia sinensis*]	ETR2
TRINITY_DN29020_c0_g1	Transcription factor bHLH19 [*Camellia lanceoleosa*]	BHLH19
TRINITY_DN292408_c0_g1	putative xyloglucan endotransglucosylase/hydrolase protein 23 [*Vitis vinifera*]	-
TRINITY_DN10100_c0_g1	hypothetical protein RHMOL_Rhmol03G0177000 [*Rhododendron molle*]	ARR17
TRINITY_DN10128_c0_g1	Protein SHORT-ROOT [*Camellia lanceoleosa*]	SHR
TRINITY_DN10353_c4_g1	hypothetical protein VitviT2T_017352 [*Vitis vinifera*]	XTH22
TRINITY_DN1048_c0_g1	serine/threonine-protein kinase BSK3-like [*Cornus florida*]	BSK7
TRINITY_DN1048_c0_g2	serine/threonine-protein kinase BSK5-like [*Diospyros lotus*]	BSK5
TRINITY_DN10571_c3_g1	probable carboxylesterase 15 [*Camellia sinensis*]	CXE15
TRINITY_DN10573_c0_g1	transcription factor MYC2-like [*Diospyros lotus*]	MYC2
TRINITY_DN10940_c0_g1	ethylene-responsive transcription factor ERF096-like [*Mangifera indica*]	ERF095
TRINITY_DN10950_c0_g1	Phosphate transporter PHO1 [*Camellia lanceoleosa*]	PHO1
TRINITY_DN10959_c0_g1	hypothetical protein CISIN_1g023816mg [*Citrus sinensis*]	ARR4
TRINITY_DN11257_c0_g2	hypothetical protein F0562_026000 [*Nyssa sinensis*]	ARF16
TRINITY_DN11356_c1_g1	LOW QUALITY PROTEIN: auxin-responsive protein IAA27-like [*Camellia sinensis*]	IAA27
TRINITY_DN118278_c0_g1	Xyloglucan endotransglucosylase/hydrolase protein 22 [*Camellia lanceoleosa*]	XTH1

Gene id	Annotation	Name
TRINITY_DN9492_c0_g1	auxin-responsive SAUR40-like [*Olea europaea* subsp. europaea]	SAUR72
TRINITY_DN986_c0_g1	hypothetical protein L1887_57101 [*Cichorium endivia*]	GID1C
TRINITY_DN9951_c0_g1	Auxin response factor 9 [*Camellia lanceoleosa*]	ARF9
TRINITY_DN39297_c0_g1	protein SCARECROW-like [*Pistacia vera*]	SCR
TRINITY_DN40703_c0_g1	auxin-responsive protein SAUR71-like [*Actinidia eriantha*]	SAUR71
TRINITY_DN4089_c0_g2	ETHYLENE INSENSITIVE 3-like 3 protein isoform X2 [*Camellia sinensis*]	EIL3
TRINITY_DN4130_c0_g1	hypothetical protein LOK49_LG10G00575 [*Camellia lanceoleosa*]	PHO1-H1
TRINITY_DN4322_c0_g1	auxin-responsive protein SAUR71-like [*Camellia sinensis*]	SAUR72
TRINITY_DN4445_c2_g1	Scarecrow-like protein [*Actinidia chinensis* var. chinensis]	SCL34
TRINITY_DN45837_c0_g1	hypothetical protein RHSIM_Rhsim11G0161000 [*Rhododendron simsii*]	CYCD3-1
TRINITY_DN45899_c0_g1	xyloglucan endotransglucosylase protein 7-like [*Cornus florida*]	XTH25
TRINITY_DN51052_c0_g1	hypothetical protein PVAP13_7NG419100 [*Panicum virgatum*]	DDB_G0282895
TRINITY_DN5158_c0_g1	protein TIFY 10B-like [*Rhododendron vialii*]	TIFY10A
TRINITY_DN5172_c0_g2	Ethylene-responsive transcription factor 1B like [*Actinidia chinensis* var. chinensis]	ERF.C.3
TRINITY_DN5295_c0_g1	hypothetical protein F0562_034388 [*Nyssa sinensis*]	AHK4

Gene id	Annotation	Name
TRINITY_DN5386_c0_g1	hypothetical protein RHSIM_Rhsim03G0226700 [*Rhododendron simsii*]	TIFY10A
TRINITY_DN16306_c0_g1	Receptor like protein kinase S.2 [*Camellia lanceoleosa*]	LECRKS2
TRINITY_DN16882_c0_g1	probable carboxylesterase 15 [*Camellia sinensis*]	CXE15
TRINITY_DN17138_c0_g1	Cyclin-D3-1 like [*Actinidia chinensis* var. chinensis]	CYCD3-3
TRINITY_DN17431_c2_g1	Serine/threonine-protein kinase SAPK10 [*Ananas comosus*]	SRK2E
TRINITY_DN177750_c0_g1	protein DOG1-like 4 [*Camellia sinensis*]	DOGL4
TRINITY_DN1790_c2_g1	hypothetical protein F0562_015535 [*Nyssa sinensis*]	BOP
TRINITY_DN181874_c0_g2	unnamed protein product [Lactuca virosa]	PUB1
TRINITY_DN18329_c0_g1	hypothetical protein GOBAR_DD09366 [*Gossypium barbadense*]	BAK1
TRINITY_DN14093_c0_g1	hypothetical protein HYC85_006434 [*Camellia sinensis*]	NSP2
TRINITY_DN1446_c5_g1	BRASSINOSTEROID INSENSITIVE 1-associated receptor kinase 1 [*Rosa chinensis*]	BAK1
TRINITY_DN14517_c0_g1	ethylene-responsive transcription factor 1B-like [*Camellia sinensis*]	ERF1B
TRINITY_DN14688_c0_g1	scarecrow-like protein 21 [*Camellia sinensis*]	CIGR1
TRINITY_DN15733_c0_g1	Transcription factor HHO3 [*Sesamum alatum*]	HHO3
TRINITY_DN15742_c0_g1	DELLA protein RGL1 [*Camellia lanceoleosa*]	D8

Supplementary table S4 selected reaction monitoring conditions for protonated or deprotonated plant hormones([M+H]+or[M-H]-)

Name	Polarity	Parent ion (m/z)	daughter ion (m/z)	De-clustering voltage (V)	Collision energy (V)
ACC	+	102.0	56.0*/84.0	40	19/42
ABA	-	263.1	153.0*/204.2	-60	-14/-27
GA1	-	347.0	259.3*/273.1	-25	-28/-16
SA	-	137	93.3*/65	-50	-20/-39
T-zeatin	+	220.1	136*/202.1/148.1/185.1	87	22/16/19/22

Note: Those marked with * are quantified ions

Supplementary table S5 Primers used for quantitative real-time PCR (qRT-PCR) analysis

**Gene id**	**Gene name**	**Forward primer (5′-3′)**	**Reverse primer (3′-5′)**
TRINITY_DN839_c0_g1	ADH2	TCTCAGCCACTTGTTTGGATAG	GGGACACCAAGAACTAGCATTA
TRINITY_DN8238_c4_g1	PDC1	GGGCAGAGGAGCGTTATATTC	ATCAACAAGGCCAGTGTAGTC
TRINITY_DN6720_c0_g2	PYL4	CTACGTCTCTTCACCTTCACAG	GGGATTGTTTGTGGCGATTAC
TRINITY_DN18929_c0_g1	SAUR51	CCCAGAAGCAGAGGTTTGT	TTCGCTATTGAAGCCGTACTC
TRINITY_DN4331_c0_g1	DK-ACO1	TAGCGCATCAAACTCCATACTC	CACTGGGAAGGTCTCCATTTC
TRINITY_DN8398_c0_g1	UBA2C	CTTCCATTCGATGTGGGTTAATG	CTGTTTCCACCTATCCCTTCTC
TRINITY_DN18925_c0_g1	UBA2C	GGTCGAATGGCTGTTTGTAATC	CAGCATCTCACTTGTCGTATCT
TRINITY_DN4322_c0_g1	SAUR72	CACTCGGCGATCAGAATCAT	TTTCGGCGACACAAGAGTATAG
GAPDH		GAAGGTCGTCATTTCAGC	GAGCAAGGCAGTTGGTAG
